# Clover root exudate produces male-biased sex ratios and accelerates male metamorphic timing in wood frogs

**DOI:** 10.1098/rsos.150433

**Published:** 2015-12-02

**Authors:** Max R. Lambert

**Affiliations:** School of Forestry and Environmental Studies, Yale University, 370 Prospect Street, New Haven, CT 06511, USA

**Keywords:** endocrine disruption, phytoandrogen, phytoestrogen, rhizosphere

## Abstract

In amphibians, abnormal metamorph sex ratios and sexual development have almost exclusively been considered in response to synthetic compounds like pesticides or pharmaceuticals. However, endocrine-active plant chemicals (i.e. phytoestrogens) are commonly found in agricultural and urban waterways hosting frog populations with deviant sexual development. Yet the effects of these compounds on amphibian development remain predominantly unexplored. Legumes, like clover, are common in agricultural fields and urban yards and exude phytoestrogen mixtures from their roots. These root exudates serve important ecological functions and may also be a source of phytoestrogens in waterways. I show that clover root exudate produces male-biased sex ratios and accelerates male metamorphosis relative to females in low and intermediate doses of root exudate. My results indicate that root exudates are a potential source of contaminants impacting vertebrate development and that humans may be cultivating sexual abnormalities in wildlife by actively managing certain plant species.

## 1. Introduction

Human activities like agriculture and suburbanization are associated with altered frog sex ratios at metamorphosis [1,2]. Sex ratio biases and other sexual abnormalities are hypothesized to be due to pesticides, pharmaceuticals, or other wastewater contaminant exposure [3–7]. With a focus on synthetic chemicals, minimal attention has been given to natural chemicals, such as phytoestrogens produced by plants. Analyses of individual phytoestrogens indicate that phytoestrogens readily impact hormone pathways important in vertebrate sexual development [8]. Despite this, we know little about how phytoestrogens enter waterways or what the biological effects of relevant phytoestrogen mixtures are [9].

**Figure 1.**
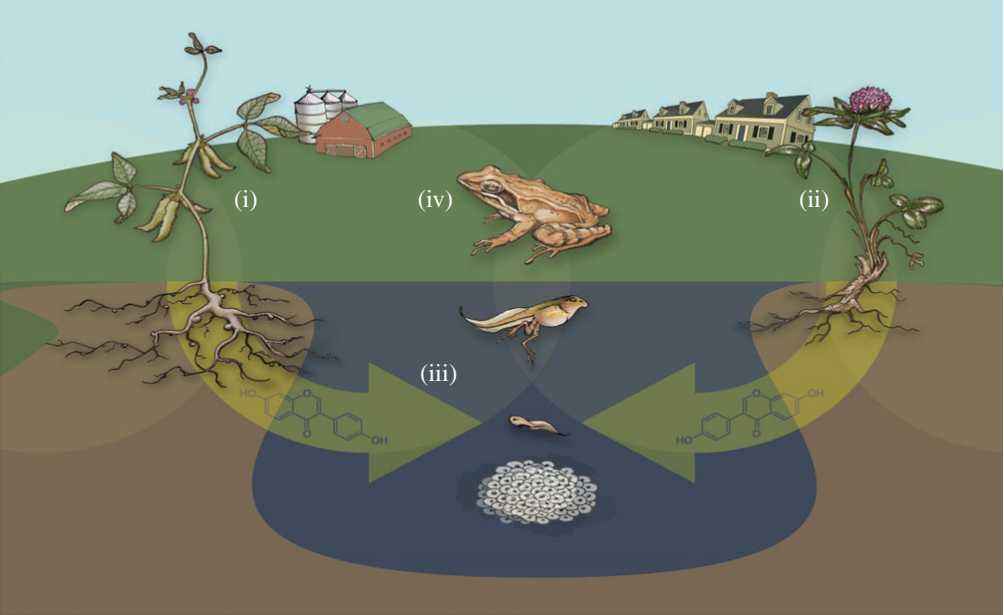
Conceptual diagram illustrating parallel impacts from plants like (i) agricultural soy or (ii) clover in suburban yards on developing (iii) and adult (iv) amphibians from root exudates. These exudates are hormonally active and hypothetically transit through the soil into aquatic ecosystems, impacting amphibian development. Designed by Monte Kawahara.

Phytoestrogens are ubiquitous in waterways from agricultural [9–11] and urbanized [2,12] landscapes. Recent evidence shows that the diversity of phytoestrogens in suburban ponds is positively correlated with the amount of surrounding anthropogenic vegetation, like lawns [2]. Interestingly, the presence of phytoestrogens in suburban ponds is also associated with abnormal frog sex ratios [2]. Phytoestrogens are assumed to originate from run-off of water over plant above-ground biomass [11]. However, root exudation (figure 1) presents an alternative mechanism for phytoestrogen contamination [2]. Phytoestrogens with known oestrogenic properties [13] are released in the chemical slurry exuded from living plant roots [14]. Functionally, root exudate phytoestrogens are used for allelopathy and inducing symbiotic mycorrhizal fungi growth [15,16]. Phytoestrogenic root exudates can become concentrated in soil [9,10] and can hypothetically transit into waterways.

Legume roots are remarkably oestrogenic, producing an equivalent of 4000 pg oestradiol (E2) per mg of plant fresh weight [17]. For reference, ovulating rats produce approximately 2.2 pg E2 per mg plasma [18]. Importantly, *in vitro* root oestrogenic activity can be two orders of magnitude more oestrogenic than above-ground plant tissues [17], indicating that root exudates may be a more likely source of environmental phytoestrogen contamination than above-ground tissues. No one has yet tested whether root exudates are potent enough to impact vertebrate development.

This experiment is motivated by recent evidence that phytoestrogen diversity (e.g. daidzein, formononetin, coumestrol) in suburban backyard ponds is associated with deviant frog sex ratios [2]. Because the diversity of phytoestrogens, rather than phytoestrogen concentrations, was positively associated with an increasing proportion of females in metamorphic frog sex ratios, my goal was to evaluate how a natural cocktail of phytoestrogens might potentially affect tadpole development. Here, I examine the impacts of root exudates from red clover (*Trifolium pratense*), a legume, on sex ratios and somatic development (metamorphic rate and mass-at-metamorphosis) of wood frogs (*Rana sylvatica*=*Lithobates sylvaticus*). I chose red clover because it is a common component of suburban yards and agricultural fields [15] where aqueous phytoestrogens have been detected and is a plant species well known to produce phytoestrogens like daidzein, formononetin, biochanin A and genistein [10], even in its roots [19].

## 2. Methods

### 2.1 Plant growth

**Figure 2.**
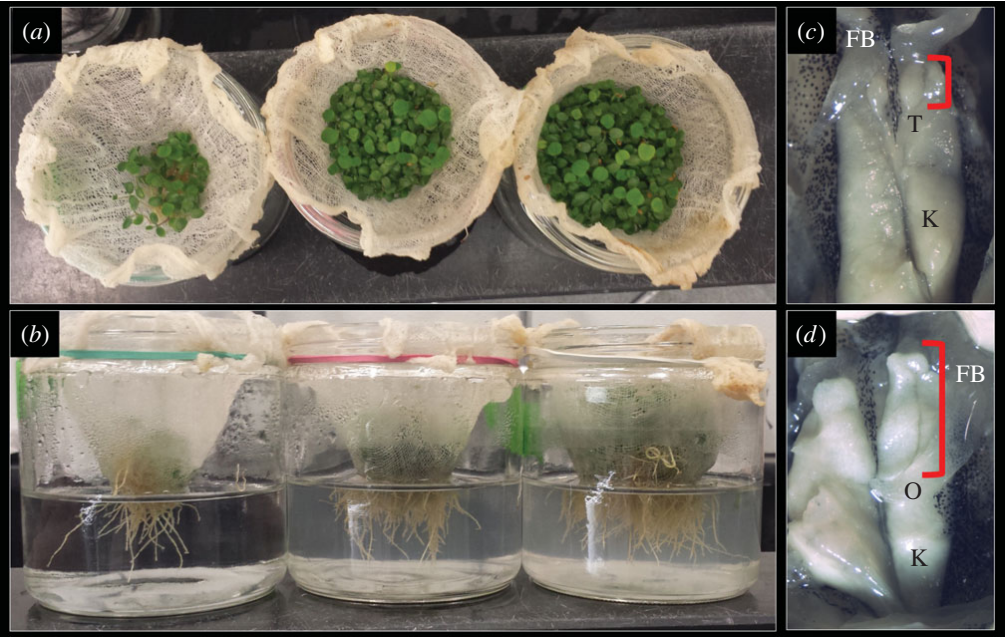
Images of experimental red clover (*Trifolium pratense*) seedling vegetative (*a*) and root (*b*) tissues grown hydroponically at three densities from left to right. Tadpoles exposed to root exudate were sexed by gross gonadal morphology as either males (*c*) or females (*d*). Red brackets indicate the length of the gonad (testes—T; ovary—O). Also represented are fat bodies (FB) and kidneys (K).

Here, I test whether root exudate impacts metamorphosing frog sex ratios and somatic developmental rates. I focus on seedling red clover (*Trifolium pratense*), a species known to produce phytoestrogens, both in laboratory [19] and field [11,20] environments and which is common both in agricultural areas [11] and suburban yards [21]. I reared red clover plants at three densities: low, medium and high (figure 2*a*,*b*). Low densities were approximately 50 seeds (0.12 g seeds), medium densities were approximately 500 seeds (1.22 g), and high densities were approximately 1000 seeds (2.44 g). I weighed 12 vials of each seed density (36 seed vials total). For surface sterilization, I soaked seeds in 95% ethanol for 5 min followed by bleach (8% NaOCl) for 10 min and then five rinses with distilled water. I then left the seeds in 300 ml of distilled water for 48 h to germinate. At the end of 48 h, I laid seeds on cheese cloth I moistened with distilled water. I laid each piece of cheese cloth in a jar filled with 200 ml of distilled water so that the seeds were just below the surface of the water. Six days later, I turned fluorescent lights on above each set of seeds. I replaced the water every 2 to 3 days and changed water for all plants of a given age on the same day. Seven days after the lights were turned on, I extracted root exudates from each jar of seedlings by removing the cheese cloth and seedlings from their jar, rinsing the jar thoroughly with distilled water, dampening the cheese cloth with distilled water and replacing the cheese cloth and seedlings into 200 ml of fresh distilled water such that only the roots were below the surface of the water. After 24 h, I pooled exudate from each plant density into separate 2000 ml glass beakers. For each dosing, I always used clover seedlings that were two weeks old. The first tadpole dosing used root exudate from clover seedlings that began germinating on 5 April 2014, were laid on cheese cloth and set in water on 7 April 2014, were under fluorescent lights beginning 13 April 2014 and which began root exudate extraction on 20 April 2014.

### 2.2 Egg collection tadpole rearing

On 9 April 2014, I collected two freshly laid wood frog egg masses from a single pond (41.320662, −72.992365) in the Yale Natural Preserve, New Haven, CT, USA, and maintained them in a glass container in an incubator between 4 and 6°C until 13 April 2014 at which point I placed eggs in glass containers in a temperature-controlled room at approximately 19°C. On 21 April 2014, all larvae were free-swimming and resorbed gills (Gosner stage 25) [22], at which point I randomly allocated individuals from each egg mass to each clover treatment or a distilled water control treatment. Jars contained 300 ml reconstituted distilled water (see below) and I immediately began dosing each tadpole with 40 ml of treatment solution. The final volume for each tadpole jar was 340 ml.

I maintained tadpoles individually in unique glass jars [23] with 300 ml of water. Maintaining tadpoles singly eradicates pseudoreplication. For water, I used distilled water that was allowed to de-gas and reach room temperature overnight in stainless steel pots. To maintain osmolarity similar to field conditions (i.e. specific conductance of approx. 100 μS), I added 0.06 g aquarium salt per litre of distilled water. Every Monday and Friday of the experiment, I either pipetted tadpoles into glass pipettes when at early developmental stages or gently poured the water and more-developed tadpole onto a stainless steel strainer, rinsed the jar with a fast stream of distilled water, refilled the jar with new water and fed the tadpoles. I fed tadpoles twice weekly, feeding each animal approximately 1/10 of its body weight per day such that I stocked each tadpole jar approximately 3/10–4/10 of its bodyweight twice a week. I fed tadpoles a 4:1 mixture of rabbit chow and fish food by mass. Because alfalfa contains phytoestrogens [24], I used Kaytee^TM^ Premium Alfalfa Free Timothy Fiber rabbit chow (13.0% crude protein, 1.5% crude fat, 25.0% crude fibre). For fish food, I use TetraFin Goldfish Crisps (44.0% crude protein, 13.0% crude fat, 2.0% crude fibre).

I checked tadpoles daily for health. I euthanized all animals on the day they reached metamorphosis (i.e. when both forelimbs emerged; Gosner stage 42) [23,25] with an overdose of MS-222. After 2 days of no additional tadpoles metamorphosing, I euthanized remaining tadpoles. I immediately measured metamorph mass and fixed the carcass in Bouin’s solution. I then sexed each individual (figure 2*c*,*d*) under a Leica MZ125 dissecting microscope and photographed gonads with a TSView7 camera. Wood frog metamorphs can easily be sexed by gross gonadal morphology at metamorphosis by observation of immature, lobed ovaries or unpigmented, smooth testes [6,25,26].

### 2.3 Statistical analyses

I asked three primary questions with these data. First, do metamorph sex ratios differ from parity when tadpoles are exposed to clover root exudate? Second, does clover affect sex-specific metamorphic timing? Third, is metamorph mass influenced by clover root exudate? I used the program R (v. 3.1.1, R Core Team) for all analyses. For all models, if block was not significant I removed it from the model.

For the first question, I used *χ*^2^ analyses to determine whether sex ratios in the control and three clover treatments different from equal proportions of males and females.

For the second question, I used linear models (‘lm’ function in R) to model whether males and females differed in metamorphic rates among treatments. I modelled metamorphic day initially as a fully saturated model incorporating a three-way interaction between metamorph sex, clover dose and block. I iteratively removed non-significant terms from the model until all terms were significant. Lastly, for each sex separately I used a linear model and Tukey’s *post hoc* tests with the mcp function in the R package ‘multcomp’ to determine if metamorphic timing varied among treatments.

For the third question, I similarly used a linear model to analyse whether metamorph mass varied as a function of clover treatment, time-to-metamorphosis, genotype, sex and block.

## 3. Results

All metamorph specimens are permanently catalogued with a unique ‘MRL’ tag number (see dataset) at the Yale Peabody Museum of Natural History along with tail and liver tissue samples stored in RNAlater (Qiagen).

### 3.1 Non-metamorphosing tadpoles

Seven animals, one control and two from each clover treatment, were excluded from analyses.

Only one tadpole (MRL 070) died during this experiment. This tadpole was in the low-dose treatment and died on 26 April 2014, 3 days after the experiment started.

Three tadpoles, one from the control treatment (MRL 229) and two from the medium-dose treatment (MRL 227, MRL 228), were arrested in development (Gosner stages 25–36) and never reached metamorphosis. One high-dose tadpole (MRL 199) developed one forelimb but not the second; this tadpole was maintained in this condition for four days at which point it was euthanized. This tadpole’s sex was used in sex ratio analyses but no other analyses. The kidney–gonad complexes from one low-dose (MRL 118) and one high-dose (MRL 183) metamorph were damaged during dissection and could not be sexed and these metamorphs were excluded from analyses.

### 3.2 Sex ratios

**Figure 3.**
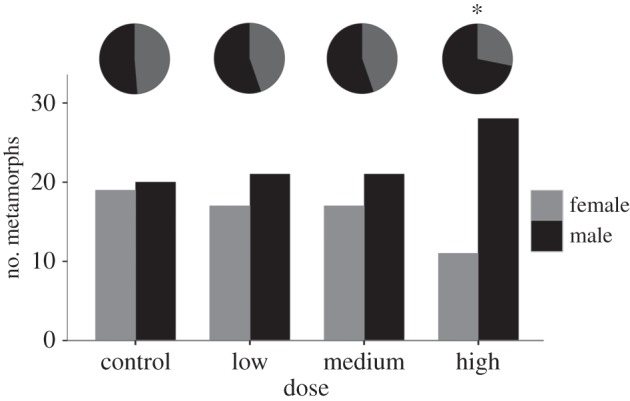
Counts of male and female metamorphs in each root exudate treatment and sex ratio pie charts. Asterisk indicates significant deviation from parity.

In the control treatment, there were 20 males and 19 females (48.7% female, *χ*^2^=0.026, *p*=0.87). In the low treatment, there were 21 males and 17 females (44.7% female, *χ*^2^=0.421, *p*=0.52). In the medium treatment, there were 21 males and 17 females (44.7% female, *χ*^2^=0.421, *p*=0.52). The high-dose treatment exhibited a significant male bias (figure 3) with 28 males and 11 females (28.2% females, *χ*^2^=7.41, *p*=0.007).

### 3.3 Sex-specific metamorphic timing

**Figure 4.**
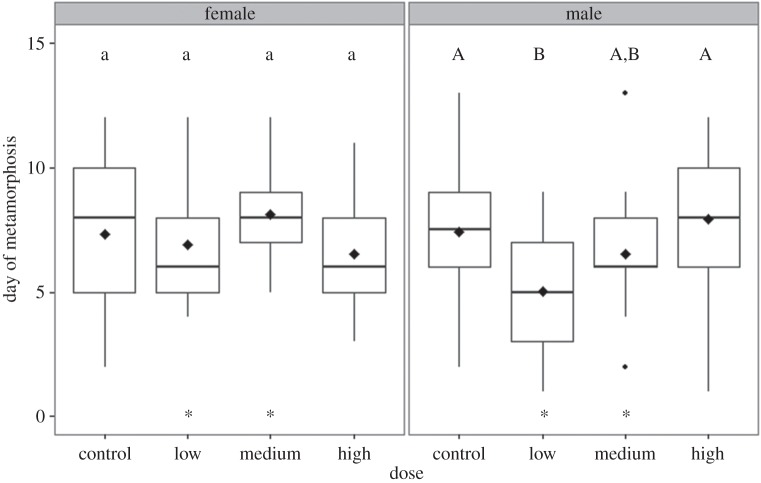
Boxplots of metamorphic timing over 13 days for both sexes in each treatment. In each box, bold lines represent the median while black diamonds represent the mean. The boxes are the first and third quartiles while the vertical lines are the highest and lowest value within the interquartile range. Letters above boxes indicate Tukey’s *post hoc* comparisons; lower case letters for females and upper case letters for males indicate separate analyses for each sex. Asterisks at the bottom of each panel indicate significant pairwise differences between males and females within a given treatment. Males from the low and medium treatments metamorphosed faster than females from the same treatments. This is in part due to accelerated metamorphic rates of males from these two treatments compared with the control males.

Clover root exudate impacted metamorphic timing, particularly for males (figure 4). The final model had a significant clover dose by sex interaction (*p*=0.02) and a significant block effect (*p*<0.001). Because of the significant interaction, I ran individual models for each treatment. Controlling for block, males and females did not differ in metamorphic rates in the control or high-dose treatments (both *p*>0.05). In the low-dose treatment, males metamorphosed faster than females (*p*=0.01); the final model included a significant block effect (full model *R*^2^=0.34, *p*=0.001, *F*=5.699_4,33_). In the medium-dose treatment, block was not significant (*p*>0.05) and was removed from the model; like the low-dose treatment, males metamorphosed faster than females (*R*^2^=0.11, *p*=0.02, *F*=5.472_1,36_).

For males, the linear model comparing day-of-metamorphosis found no effect of genotype (*p*>0.05) but, controlling for block, there were differences among treatments (*R*^2^=0.23, *p*<0.001, *F*=5.391_6,83_). Tukey’s *post hoc* tests indicated that low-dose males metamorphosed earlier than control males (*p*=0.04) and high-dose males (*p*=0.003) but not medium-dose males (*p*>0.05). No other pairwise comparisons were significant.

For females, the linear model found no effect of genotype on day-of-metamorphosis and there were no differences in day-of-metamorphosis among treatments even when controlling for block (*p*>0.05).

The average day-of-metamorphosis in the control group was 7.4 (±0.62) days for males and 7.3 (±0.65) days for females, in the low group it was 5.05 (±0.49) days for males and 6.88 (±0.62) days for females, in the medium group it was 6.5 (±0.53) days for males and 8.12 (±0.38) days for females, and in the high group it was 7.93 (±0.54) days for males and 6.55 (±0.71) days for females.

### 3.4 Mass

For females, the model found that day-of-metamorphosis, genotype, and block were all non-significant (*p*>0.05). A Tukey’s *post hoc* test found no significant differences among treatments (all *p*>0.05), though low-dose females were trending towards being lighter than high-dose females (*p*=0.09).

For males, the model found no effect of block or genotype (both *p*>0.05) but found a significant effect of day-of-metamorphosis (*p*=0.03). A Tukey’s *post hoc* test when controlling for day-of-metamorphosis found no significant differences among treatments, though high-dose males were trending towards being heavier than control males (*p*=0.067).

## 4. Discussion

Plant root exudates can cause sex ratio biases in frogs, implying sex reversing effects. The magnitude and type of effects reported here are comparable with responses seen in frogs exposed to pesticides [5] and pharmaceuticals [4,6]. This is the first *in vivo* demonstration that root exudates impact vertebrate sexual and somatic development.

Root exudation is an unaccounted for source of contamination that may be contributing to abnormal sexual development in wildlife. With 9.5 million ha of lawn [27] and 34.3 million ha of soy [28] planted in the United States, a combined area larger than California, the impacts of hormonally active root exudates could be pervasive. Many legumes are planted to enhance soil quality or are important crops [15,21]. Management for such plants could contaminate aquatic habitats with phytochemicals capable of interfering with typical vertebrate development. The results presented here indicate that the impacts of root exudates on aquatic organisms deserve more attention.

While little is known about the physiological basis of sexual differentiation in wood frogs, some recent evidence suggests that aromatase expression is elevated in developing female *R. sylvatica* relative to developing males [29], indicating that oestrogens are probably important for ovarian differentiation while androgens are probably important for testicular differentiation. Most hormonally active chemicals studied produce female-biased sex ratios in frog metamorphs [4–6]. Interestingly, wood frog sex ratios here were biased towards males. One explanation for this is that some taxa have unexpected masculinizing responses to otherwise feminizing chemicals, where oestrogen exposure produces all males rather than all females [30]. If root exudates are oestrogenic, wood frogs may counterintuitively become masculinized. Furthermore, different species exposed to the same oestrogenic chemical can differ in their physiological responses [8]. Further work is needed to understand whether the masculinizing effect seen here is species-specific and if root exudates are more typically feminizing.

Alternatively, clover exudate may be more androgenizing than oestrogenic. Limited evidence suggests that plant compounds thought to be oestrogenic can have net androgenic effects [31]. The chemical mixture in root exudates may therefore, in whole, be predominantly androgenic. Furthermore, the diversity of hormonally active phytochemicals exhibit an array of agonistic and antagonistic effects on oestrogen and androgen receptors [32], indicating that chemicals in clover root exudate may alternatively impose an anti-oestrogenic influence on wood frogs rather than a direct androgenic effect. The physiological impact of chemical mixtures produced by plants on vertebrate sexual development may also vary between plant species [33]. More work is certainly needed to understand the effects of chemical mixtures produced by a variety of plant species on vertebrate development. The results here indicate that developing an understanding of how different plant species impact the physiology and development of animals will be a fruitful research direction. In particular, we need to explore whether root exudates, as well as the chemical mixtures produced by other plant organs, have similar hormonal impacts throughout the vertebrate body (e.g. gonads versus brain).

The other pattern emerging from these data is sex-specific metamorphic onset. Specifically, in the low- and medium-dose treatments, males metamorphosed faster than females. This result appears to be due to males metamorphosing earlier when exposed to root exudate, rather than females metamorphosing more slowly (figure 4). Recent work demonstrated that female wood frog tadpoles on a high-food diet exhibited faster metamorphic rates than males; a low-food diet increased this difference [26]. Another study on wood frogs found that, in control treatments, females metamorphosed later than males but that exposure to a herbicide eliminated sex differences in metamorphic timing [25]. By contrast, my results show that females metamorphose later than males only when exposed to clover root exudate. Together, my current study and these two prior studies indicate that environmental factors can have significant impacts on the relative developmental timing between sexes. Because males and females reared in the absence of root exudate did not differ in developmental rates, it is likely that differences in development rates between the sexes may not be innate but rather due to exogenous cues. One study demonstrated that short-term exposure to the phytoestrogen genistein can inhibit tail resorption in metamorphosing bullfrogs (*Rana catesbeiana*) via the thyroid hormone pathway [34]. This means that outside of influencing sex steroid regulation and sexual differentiation, phytochemicals may have a larger effect on other hormonal pathways than is generally appreciated. The extent to which phytochemicals impact metamorphosis and other physiological processes is sex-specific is currently an open arena for research. Furthermore, whether developmental rates differ among sexes in wild populations is unknown. Because variation in larval developmental rates has consequences for terrestrial stages and fitness [35], differences in metamorphic timing between the sexes may lead to sex-specific variation in carryover effects.

This experiment was directly motivated by evidence showing that deviant frog metamorph sex ratios are associated with the presence of a diversity of phytoestrogens in suburban ponds [2]; this same diversity of phytoestrogens is also commonly found in the root exudates of plants, including clovers [14–16]. The results here should be interpreted with some caution, however, as it is currently unclear what the relationship may be between the concentrations of chemicals experienced by tadpoles in this experiment relative to the concentration of chemicals present in the environment due specifically to root exudates. Furthermore, the hypothesis that phytoestrogen mixtures in root exudates contaminate surface waters is not restricted to production by only red clover. Rather, it is likely that a diversity of plants contribute phytoestrogens to waterbodies. Understanding the contribution of phytoestrogens, from both clovers as well as other plant species, to surface waters will necessitate further field experimentation. Regardless, this experiment questioned whether the amalgamation of chemicals present in root exudates was capable of impacting tadpole development; the results from this experiment indicate that root exudates can certainly affect tadpole sexual and somatic development.

Plant root exudates can impact both sexual and somatic development in amphibians. My study highlights an important connection between plant ecology and vertebrate developmental biology. Without discrediting the impacts of synthetic chemicals, plant chemicals may be a contributing cause of abnormal sexual development in agricultural and suburban environments. Future work should be targeted at understanding the relative impacts of plant chemicals on vertebrate development in different ecological contexts.

## Supplementary Material

‘Clover_Experiment_Data_RoySocOpen_Lambert _ 01 November 2015’ is the data file

## Data accessibility

Raw data are in the electronic supplementary material.
